# Trends in Sudden Cardiac Death Related Mortality in Adults in the United States: A CDC WONDER Database Analysis, 1999–2020

**DOI:** 10.1002/clc.70180

**Published:** 2025-07-15

**Authors:** Riya Bhagwan, Rayyan Nabi, Shree Rath, Sohaib Aftab Ahmad Chaudhry, Shehdev Meghwar, Diya Rathi, Sandhiya Prem Kumar, Neha Bhagwan Das, Peter Collins, Hasan Ahmad, Raheel Ahmed

**Affiliations:** ^1^ Dow University of Health Sciences Karachi Pakistan; ^2^ Islamic International Medical College Rawalpindi Pakistan; ^3^ All India Institute of Medical Sciences Bhubaneswar India; ^4^ ABWA Medical College Faisalabad Pakistan; ^5^ Liaquat University of Medical and Health Sciences Jamshoro Pakistan; ^6^ People's University of Medical and Health Sciences Nawabshah Pakistan; ^7^ National Heart and Lung Institute, Imperial college London London UK

**Keywords:** cardiovascular disease, mortality, sudden cardiac death

## Abstract

**Background:**

Sudden cardiac death (SCD) is a leading cause of mortality in the United States, with significant variations across demographic and geographic factors. This study analyzes trends in SCD‐related mortality among adults (> 25 years) from 1999 to 2020 using the CDC WONDER database.

**Methods:**

We extracted data on SCD‐related deaths (ICD‐10 code I46.1) and calculated age‐adjusted mortality rates (AAMR) per 100 000 population, stratified by sex, race/ethnicity, urbanization, and census region. Joinpoint regression was performed to estimate the annual percent change (APC) and average annual percent change (AAPC) with 95% confidence intervals (CI).

**Results:**

A total of 279 599 SCD‐related deaths were recorded from 1999 to 2020. Overall AAMR declined significantly (AAPC: −1.20%; 95% CI: −1.58% to −0.82%) until 2018, followed by a sharp increase from 2018 to 2020 (APC: +6.93%; 95% CI: +3.04% to +10.96%). Declines were most pronounced in American Indian/Alaska Native populations (−3.67%), while the highest increases post‐2018 were observed in Hispanic (+13.1%) and Asian/Pacific Islander groups (+12.3%). Urban areas experienced greater post‐2018 increases compared to rural areas. Regional disparities were evident, with the West showing the steepest rise in mortality.

**Conclusion:**

While SCD mortality declined from 1999 to 2018, a concerning reversal has emerged since 2018, particularly in specific racial/ethnic groups and urban areas. Further research is needed to investigate underlying causes, including the potential impact of COVID‐19, healthcare disparities, and lifestyle factors.

## 1 Introduction

Approximately 50% of cardiovascular mortality is due to sudden cardiac death (SCD), making it a significant global health issue. Additionally, it results in over four million deaths each year, representing one‐fifth of all deaths attributed to diseases worldwide [1, 2]. It is an unexpected and sudden death caused by a cardiac condition that happens within an hour of the symptom onset and is linked to a cardiac cause. Despite its significance and societal burden, understanding of SCD epidemiology is limited, particularly due to definitional and methodological problems [1, 3]. The leading cause of death in the United States remains cardiovascular disease, with coronary heart disease (CHD) responsible for approximately 700 000 deaths each year. According to a report by the Centers for Disease Control and Prevention (CDC), sudden cardiac death (SCD) results in about 10%–15% of all annual deaths and constitutes nearly half of all deaths related to CHD [4, 5]. SCD occurs more typically in men than in women. According to a study, women with SCD are less likely than males to have a history of cardiac disease (37% vs. 56%) [6]. Coronary artery disease (CAD) is the leading cause of SCD in the elderly, whereas among people aged 35 + , it is more often caused by structural heart disease. SCD is the main cause of mortality among young competitive athletes. Previous investigations have shown widely varying estimates of SCD incidence in young athletes, owing mostly to methodological problems [7, 8]. A previous study has shown that the mortality rate from SCD is rising among young adults in the US, specifically those aged 25 to 44 years, with significant disparities linked to racial background, population density, and geographic region [9].

Despite the high incidence of SCD in the United States, only one study has looked at death rates for those aged 25 to 44 years. Our specific goal is to investigate how biological sex, race, ethnicity, and topography affect SCD mortality among adults. Furthermore, considering the recent rise in the death trajectory, we looked at how the COVID‐19 pandemic affected the SCD mortality trend.

## 2 Methods

### 2.1 Study Setting and Population

The Centers for Disease Control and Prevention Wide‐Ranging Online Data for Epidemiologic Research (CDC WONDER) database was accessed to extract the records of SCD‐related mortality in the United States [10]. We investigated the mortality rates among adults (> 25) who died because of sudden cardiac death between 1999 and 2020. The Multiple Cause‐of‐Death Public Use record death certificates were analyzed to ensure that all cases in which SCD had been identified as a direct or underlying cause of death were included [11].

The International Statistical Classification of Diseases and Related Health Problems, 10th Revision (ICD‐10) code I46.1 was used to identify people with SCD in individuals older than 25 [12]. These codes have also been implemented in previous studies [9].

Institutional review board approval was not required for this study since the CDC WONDER database, which contains deidentified data, is publicly accessible. Additionally, this study complies with the guidelines of Strengthening the Reporting of Observational Studies in Epidemiology (STROBE).

### 2.2 Data Extraction

Multiple demographic factors, including sex, region, race/ethnicity, and urban/rural classification, were included in the extensive data set utilized for the study. There were male and female sex categories. Regions were categorized using the Census Bureau's criteria into Northeast, Midwest, South, and West. Race/ethnicities groups were characterized as Hispanic, Non‐Hispanic White, Non‐Hispanic Black, Non‐Hispanic Asian or Pacific Islander, Non‐Hispanic American Indian/Native American. This information has also been utilized in earlier studies using the WONDER database and is based on reported death certificates [11].

The National Center for Health Statistics' Urban‐Rural Classification Scheme was employed to stratify the population geographically, dividing the counties into urban (large metropolitan area;

population over a million), medium/small metropolitan area (50 000–999 999), and rural (less than 50 000) categories.

### 2.3 Statistical Analysis

We computed nationwide trends in SCD‐related mortality to assess the crude and age‐adjusted mortality rate (AAMR) per 100 000 population from 1999 to 2020, stratified by year, gender, race, and urban–rural status with a 95% confidence interval (CI). Crude mortality rates were achieved by dividing the total number of deaths associated with SCD for each relevant year by the corresponding US population. To determine age‐adjusted mortality rates, SCD‐related mortality was standardized to the US population in 2000.

Joinpoint Regression Programme (Version 5.0.2, National Cancer Institute) was used to determine the annual percentage change (APC) and its corresponding 95% confidence interval (CI) for age‐adjusted mortality rates (AAMRs) [13]. This software adjusts log‐linear regression models when temporal shifts occur to determine significant changes in age‐adjusted mortality across time. Significant increases or decreases were identified using a 2‐tailed *t*‐test with *α* = 0.05 to evaluate if the change in mortality during each period was statistically different from zero.

## 3 Results

From 1999 to 2020, there were a total of 279 599 deaths categorized as Sudden Cardiac Deaths (SCD) in the US among individuals aged 25 years to over 85 years. The results have been stratified by various factors, including Year, Gender, Race, Urbanization, and Census Region.

### 3.1 SCD Related AAMR Stratified by Year

**Figure 1:**
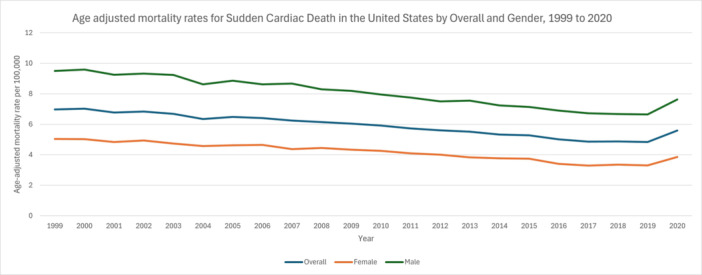
Age adjusted mortality rates for sudden cardiac death in the US by gender, 1999–2020.

From 1999 to 2009, overall age‐adjusted mortality rates (AAMR) for SCD declined at an Annual Percent Change (APC) of −1.4636 (95% CI: −1.7546 to −1.1718). This downward trend continued from 2009 to 2018 at −2.6306 (95% CI: −3.0384 to −2.2210) before reversing between 2018 and 2020, when the rate rose by 6.9295 (95% CI: 3.0411 to 10.9647). Across the full period (1999–2020), the Average Annual Percentage Change (AAPC) was −1.1992 (95% CI: −1.5780 to −0.8190) (Figure 1, Figure S5).

### 3.2 SCD Related AAMR Stratified by Gender

Among females, the AAMR declined from 1999 to 2010 at −1.5146 (95% CI: −1.8890 to −1.1387), dropped further from 2010 to 2018 at −3.3813 (95% CI: −4.1459 to −2.6105), and then rose by 7.6678 (95% CI: 1.5914 to 14.1077) between 2018 and 2020. Overall (1999–2020), the AAPC was −1.3963 (95% CI: −1.9906 to −0.7985). In males, the rate fell from 1999 to 2018 at −2.0183 (95% CI: −2.1804 to −1.8560) and increased by 5.4885 (95% CI: 0.1448 to 11.1174) from 2018 to 2020, yielding an AAPC of −1.3270 (95% CI: −1.8000 to −0.8517) (Figure 1, Table 1, Figure S1).

### 3.3 SCD Related AAMR Stratified by Race/Ethnicity

**Figure 2:**
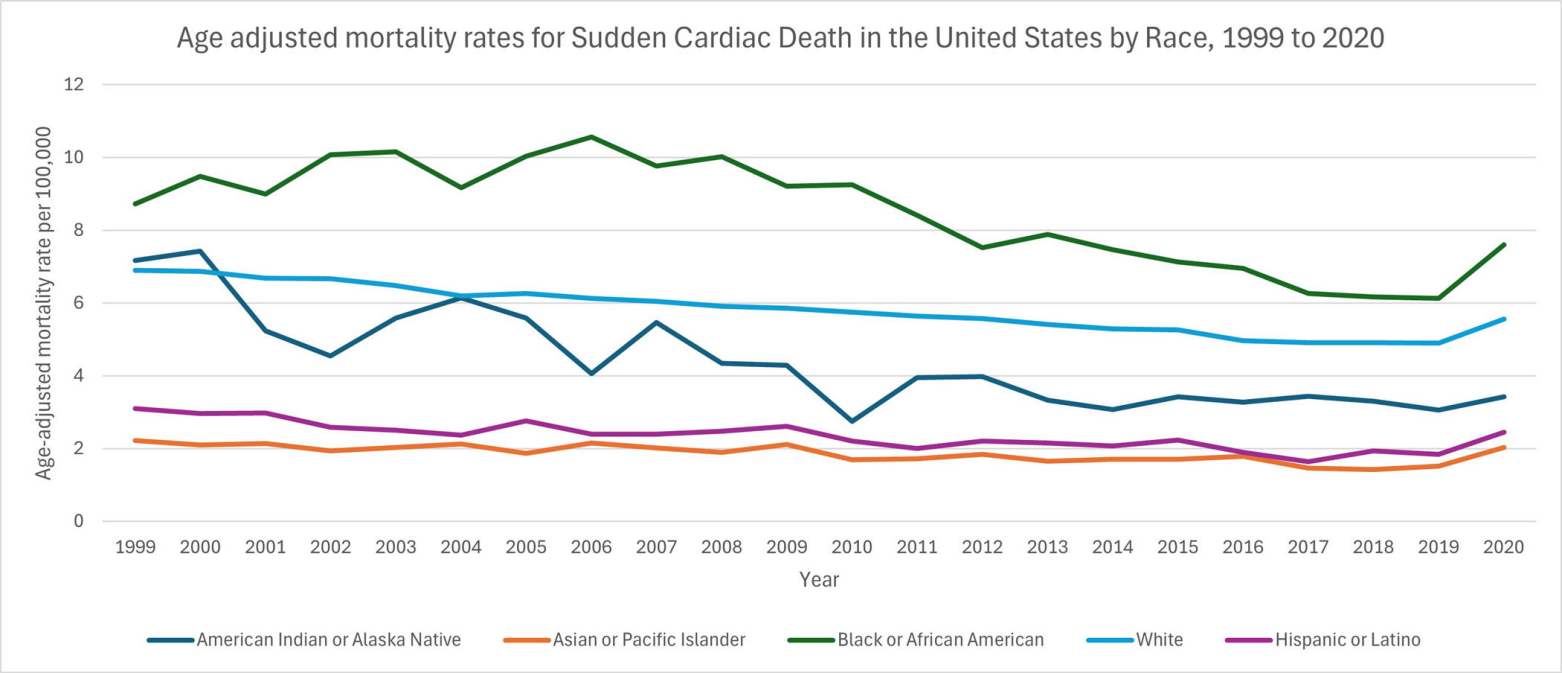
Age adjusted mortality rates for sudden cardiac death in the US by race, 1999–2020.

Among American Indian or Alaska Native individuals, the AAMR declined from 1999 to 2020 at an APC of −3.6702 (95% CI: −4.5940 to −2.7375), likewise yielding an AAPC of −3.6702 (95% CI: −4.5940 to −2.7375). In the Asian or Pacific Islander group, the AAMR dropped to −2.0491 (95% CI: −2.6570 to −1.4373) between 1999 and 2018, then rose to 12.3407 (95% CI: −3.7296 to 31.0937) from 2018 to 2020, resulting in an overall AAPC of −0.7620 (95% CI: −2.2029 to 0.7002). For Black or African American individuals, the rate increased from 1999 to 2006 at 2.3786 (95% CI: 0.5432 to 4.2474), decreased from 2006 to 2018 at −4.3927 (95% CI: −5.2147 to −3.5635), and then rose by 9.2890 (95% CI: −3.4625 to 23.7248) from 2018 to 2020. The full‐period AAPC was −0.9330 (95% CI: −2.2061 to 0.3566). Among White individuals, the AAMR declined at −1.8623 (95% CI: −1.9708 to −1.7537) from 1999 to 2018 and then rose to 5.4060 (95% CI: 1.6355 to 9.3164) from 2018 to 2020, for an AAPC of −1.1923 (95% CI: −1.5231 to −0.8603). Finally, in the Hispanic or Latino group, the rate fell at −2.5670 (95% CI: −3.2314 to −1.8980) between 1999 and 2018 before climbing at 13.1139 (95% CI: −5.2997 to 35.1079) from 2018 to 2020, resulting in an overall AAPC of −1.1723 (95% CI: −2.8128 to 0.4958) (Figure 2, Table S2, Figure S2).

### 3.4 SCD Related AAMR Stratified by Urbanization Status

**Figure 3:**
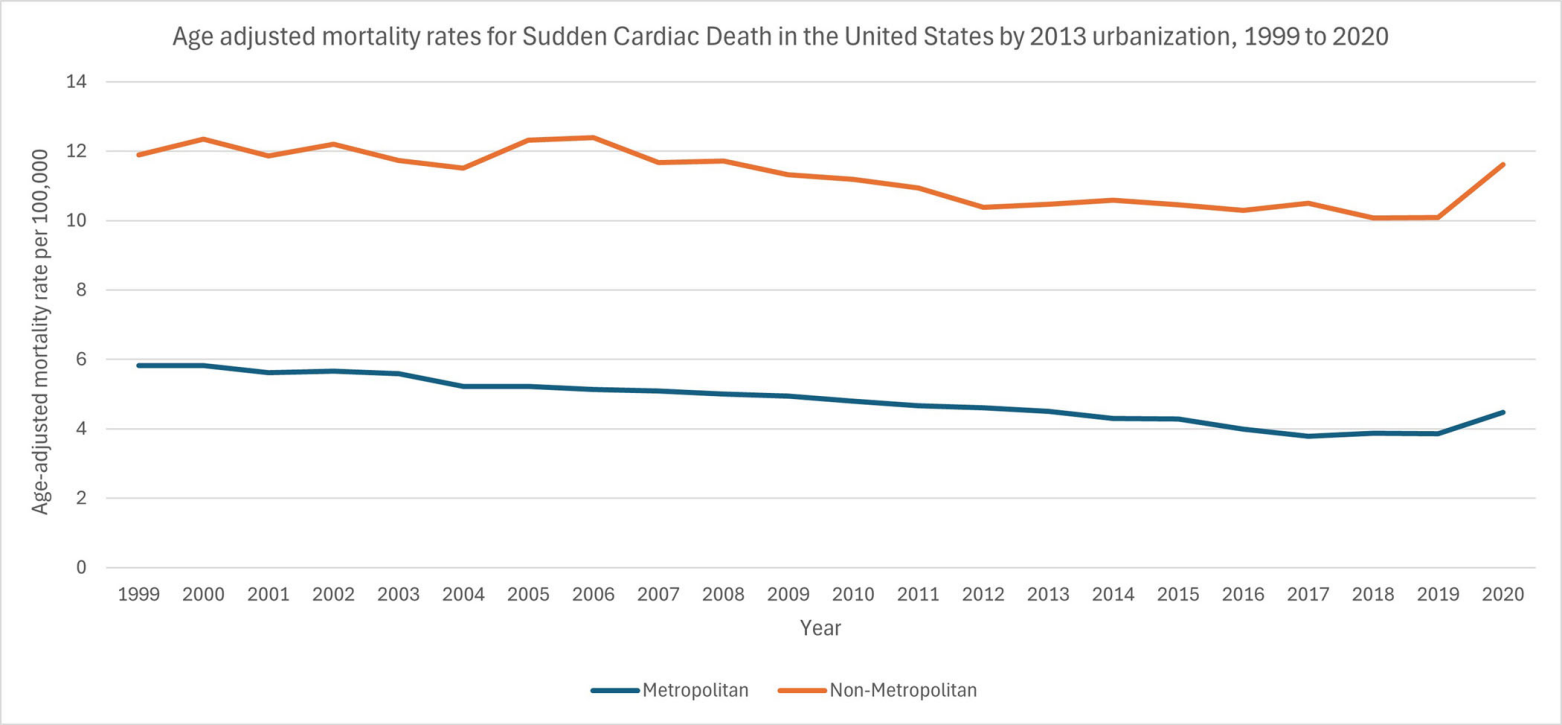
Age adjusted mortality rates for sudden cardiac death in the US by metropolitan status, 1999–2020.

In metropolitan areas, the AAMR declined at −1.8843 (95% CI: −2.0942 to −1.6740) from 1999 to 2013, continued downward at −3.7793 (95% CI: −5.1942 to −2.3433) between 2013 and 2018, and then reversed to a 8.5604 (95% CI: 3.8333 to 13.5027) increase from 2018 to 2020, resulting in an AAPC of −1.3934 (95% CI: −1.9054 to −0.8787). By contrast, in nonmetropolitan areas, the rate fell from 1999 to 2018 at −1.0908 (95% CI: −1.3657 to −0.8151) and rose modestly by 5.6076 (95% CI: −3.6229 to 15.7220) during 2018–2020, for an AAPC of −0.4716 (95% CI: −1.3064 to 0.3703) (Figure 3, Table S3, Figure S3).

### 3.5 SCD Related AAMR Stratified by Region

**Figure 4:**
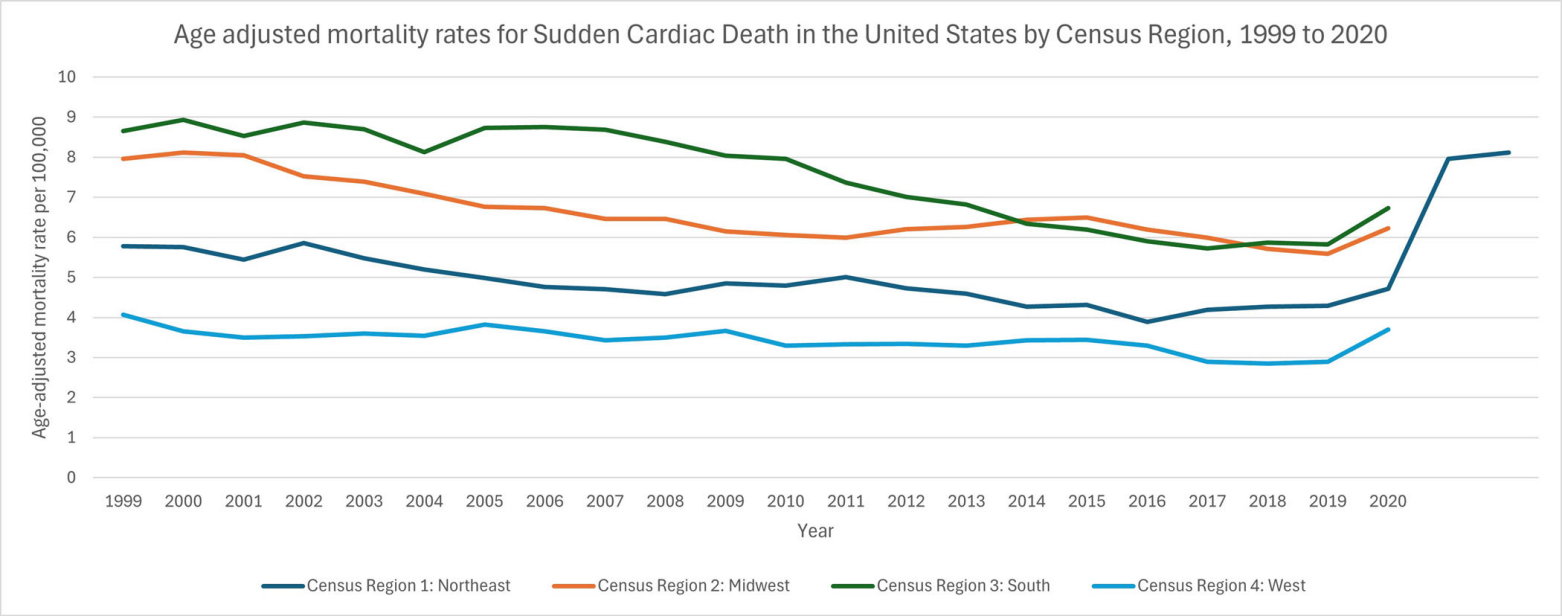
Age adjusted mortality rates for sudden cardiac death in the US by region, 1999–2020.

In the Northeast, from 1999 to 2002 the AAMR changed minimally at −0.1645 (95% CI: −3.8055 to 3.6144). It then declined from 2002 to 2007 at −4.1863 (95% CI: −6.4621 to −1.8552), rose slightly from 2007 to 2011 at 1.9676 (95% CI: −1.9180 to 6.0072), and dropped again from 2011 to 2016 at −4.3391 (95% CI: −6.6449 to −1.9764). Between 2016 and 2020, it rebounded by 3.7525 (95% CI: 1.3084 to 6.2556), yielding an overall AAPC of −1.0199 (95% CI: −2.1109 to 0.0833). In the Midwest, the rate fell from 1999 to 2010 at −2.8537 (95% CI: −3.2347 to −2.4712), increased to 1.6750 (95% CI: −0.2094 to 3.5950) between 2010 and 2015, dipped from 2015 to 2018 at −4.7008 (95% CI: −10.0925 to 1.0141), and then rose by 4.0426 (95% CI: −1.8133 to 10.2478) from 2018 to 2020, for an AAPC of −1.4212 (95% CI: −2.3929 to −0.4399). In the South, there was little change from 1999 to 2008 at −0.2780 (95% CI: −1.0362 to 0.4860), followed by a drop of −4.5670 (95% CI: −5.4439 to −3.6818) between 2008 and 2017, and a subsequent rise of 5.0837 (95% CI: 0.8473 to 9.4980) from 2017 to 2020. Across the full range (1999–2020), the AAPC was −1.4043 (95% CI: −2.1050 to −0.6986). Finally, in the West, the AAMR declined at −0.6844 (95% CI: −1.1614 to −0.2051) from 1999 to 2015, shifted to −6.3467 (95% CI: −16.9044 to 5.5525) from 2015 to 2018, and then rose by 14.0804 (95% CI: 1.9631 to 27.6377) between 2018 and 2020, yielding an overall AAPC of −0.2052 (95% CI: −2.0562 to 1.6809) (Figure 4, Table S4, Figure S4).

**Figure 5:**
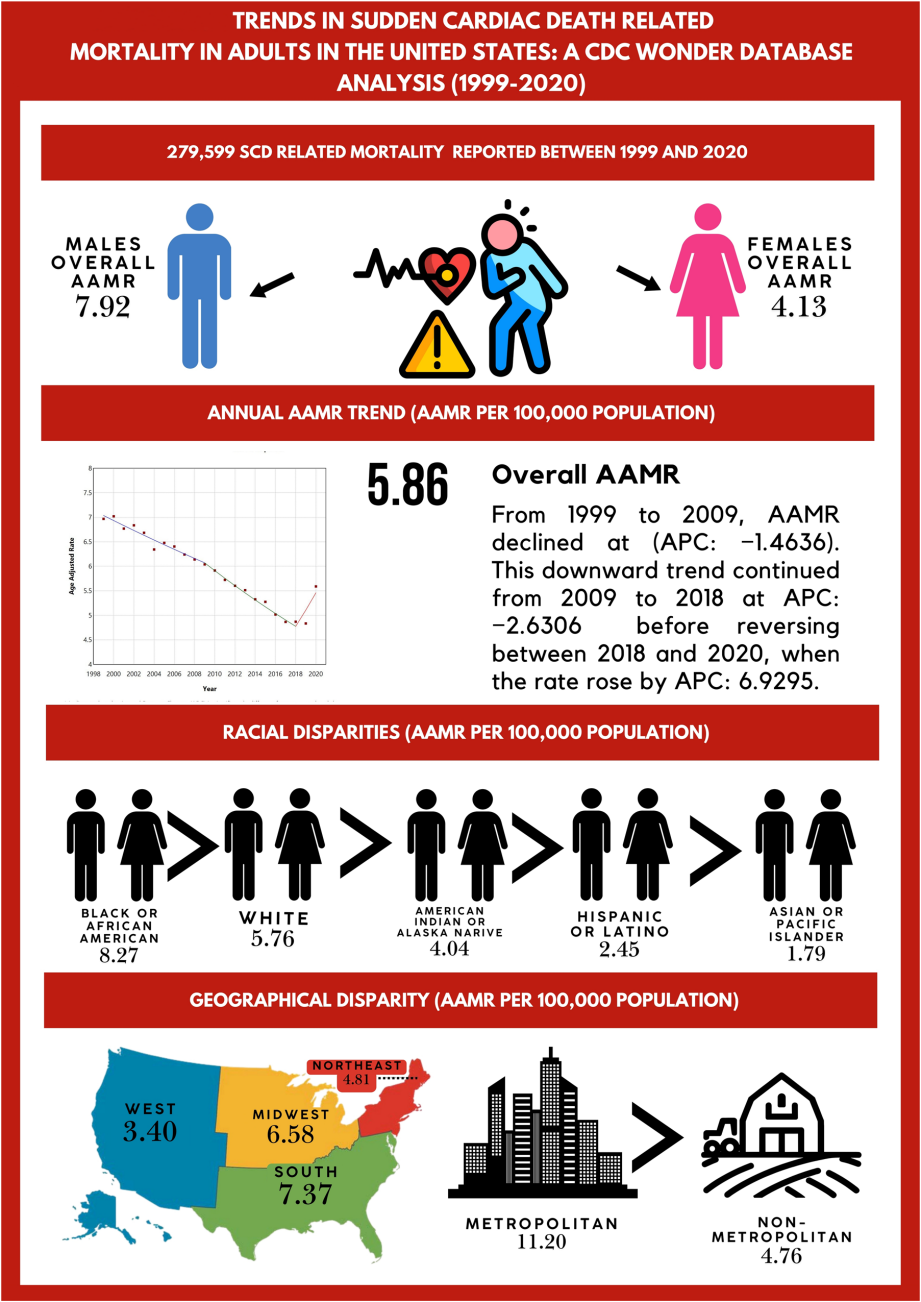
Central Illustration depicting summary of our results.

Figure 5 is a central illustration showing a summary of our findings.

## 4 Discussion

Our results concluded an overall decline in mortality among those aged due to SCD despite the steep rise in the past 2 years from 2018 to 2020. Further, a steeper incline since 2018 has been noted in females than males. Overall decline over the past two decades was highest among American Indians and Alaskan natives, while a rising trend in mortality has been noted among the other races since 2018, with the highest among Hispanics followed by Asians and Black and African Americans. Mortality has been rising among both metropolitan and nonmetropolitan regions since 2018, with a pronounced increase in metropolitan regions. A sharp incline in mortality was noted in metropolitan regions. The West region has observed the steepest incline since 2018 despite the nonsignificant trend from 1990 to 2021.

Across the years, our analysis noted a decline in mortality till 2009 and a sharper decline from 2009 to 2018, followed by a sudden rise in deaths from 2018 to 2020. This contrasts overall trends among adults aged 25–44 years, in whom mortality has been uniformly increasing since 1990 [9]. While SCD can result from a multitude of cardiac and noncardiac causes, among the elderly, mortality is pronounced due to cardiac causes, particularly degenerative heart conditions [14]. These conditions increase in prevalence with age, with nearly 62.9% of all adults > 85 years being affected with any degenerative valvular heart disease. Concurrent comorbidities like renal insufficiency can significantly increase the risk of mortality (*p* < 0.01) [15]. In 1999 across 10‐year age cohorts, the highest number of deaths were reported among those above 85, and nearly two‐thirds of these patients had an out‐of‐hospital death, elucidating the inaccessibility to emergency healthcare services [16]. From 1990 till 2018, the decline in mortality may have been due to improved emergency medical services [17], and the development of drugs offering mortality benefits like beta‐blockers and amiodarone have further enhanced the reduction [18]. However, the sudden rise in AAMRs since 2018 is concerning. Globally and in the US, the burden of cardiovascular diseases has been rising in the past 5 years [19]. Further, risk factors for cardiovascular diseases have been on the rise, one of which includes obesity. In 23 states of the US, a prevalence of over 35% has been noted of obesity, thus contributing to increased risk of CVD and SCD [20]. The rise of SCD during the COVID‐19 pandemic proposed a bidirectional relationship between both conditions. Autopsy reports of patients with SCD noted SARS‐CoV virus in the myocardium which suggests an inflammatory mechanism leading to death [21]. Infection with COVID‐19 increased the risk of mortality due to CVD by nearly three times, thus necessitating routine monitoring of the cardiovascular parameters of patients [22].

While overall mortality trends from 1990 to 2020 indicated a steeper fall in females than males, the period from 2018 to 2020 witnessed a sharper incline in deaths among females than males. A previous study described a higher prevalence of SCD in men, with more cases being ischemic in etiology. However, the same study describes a 13.7% per decade rise in ischemic SCD among women than in men (11.4% per decade) [23]. Therefore, despite higher prevalence in men, recent trends have noted a sharper increase in females. This could be accounted for by the higher risk of mortality in females following acute cardiac events than in males [24]. One cause of this increased mortality is the varied presentation in women. Multiple studies have indicated that presentation in women is often more subtle and goes unnoticed, thus resulting in delay of care [25, 26]. Additionally, a rising issue in the field of cardiovascular therapy is the gross inequity and underrepresentation of women, both in clinical trials and when formulating guidelines. The lack of personalized care and gender‐stratified evidence results in a lack of understanding of whether drugs hold similar efficacy in real‐world cases among women as they do in a trial setup [27, 28]. Another cause of the higher risk of SCD could be the higher risk of mortality at milder stages of coronary diseases. Sherpa et al. noted that women with minimal to mild coronary artery diseases had a higher risk of SCD than men [29]. Hence, the inequity in seeking treatment, the lack of personalized medicines, and the dearth of studies specific to females may account for the gross disparities.

Hispanics have noted the highest incline in mortality from 2018 to 2020. Reinier et al. described multiple factors associated with a higher risk of death, one of the highest being the presence of chronic kidney disease [30]. Correspondingly, the incidence of kidney diseases and end‐stage renal disease is nearly 50% higher in Hispanics than non‐Hispanics [31]. This higher prevalence is linked to the higher burden of diabetes and metabolic syndromes among Hispanics, which‐in part‐ directly increases the risk of SCD [32]. Rates of uninsured are higher in Hispanics, which hinders the affordability of healthcare and treatment [33]. A higher proportion of Hispanics live in poverty which further impairs affordable healthcare [34]. Closely following the Hispanics, Asian and Pacific Islanders (API) had a high rise in mortality due to SCD from 2018. Waitzfelder et al. noted APIs to have high cardiovascular mortality, particularly due to a higher prevalence of comorbidities like hypertension, metabolic syndrome, and diabetes. Obesity among pacific islanders is at 41%, which subsequently predisposes them to heart attack or stroke [35]. The COVID pandemic resulted in younger API and Hispanic patients being afflicted with SARS‐CoV‐2 than non‐Hispanic Whites [36]. Further, those who had COVID at a younger age had more chances of death due to acute heart ischemia and myocarditis as opposed to the elderly who were predisposed to respiratory pathologies [37]. African Americans noted the third highest increase in mortality due to SCD, and higher than Caucasians. Multiple studies have highlighted this disparity and suggest causes like lifestyle factors and genetics influencing the higher mortality [38, 39]. African Americans were less likely to receive permanent pacemakers that Whites and had lesser improvements that Whites on use of similar doses of beta‐blockers [40, 41]. American Indian and Alaskan Natives recorded an overall uniform decline in mortality, despite higher mortality due to cardiovascular diseases noted among them [42]. Racial misclassification of this group may have hindered true estimation, and real‐world cohorts are needed to adequately quantify burden of SCD [43].

Metropolitan regions have noted the highest incline in SCD from 2018, which may have been influenced by the growing incidence of lifestyle diseases. Obesity and diseases like diabetes are higher in urban households, further exacerbating the risk of cardiovascular deaths [44]. While rural trends are less steep than their urban counterparts, the increase in these regions may have been influenced by the response time of emergency medical services and bystander CPR [45].

By region, SCD‐related rise in AAMR was pronouncedly higher among Western regions from 2018, followed by the South and Midwest. West regions are known for their extremes of climate, particularly heat waves. It has been seen that sudden cardiac deaths are commonly associated with exposure to too hot or too cold climates, with higher temperatures contributing to 1.5% of SCD [46]. Hot temperatures exacerbate cardiac conditions as well, further increasing the risk of mortality [47]. On the other hand, cardiovascular deaths are higher in the southern region, implying a higher prevalence of cardiac SCD deaths here [48]. Northeastern regions have recorded the slowest rise in deaths since 2018, which may have been due to affordable and accessible healthcare services [49].

Our study assessed overall trends in SCD from 1990 to 2020, highlighting the rising trends since 2018. Some limitations of this study include incorrect or missing data inherent to the CDC‐WONDER database, misclassification of deaths from death certificates [50], and the lack of elucidation of risk factors and underlying causes of SCD‐related deaths. Future studies should assess the risk factors behind varying trends and attempt to subclassify SCD as due to cardiac and noncardiac causes to assess the burden of each category.

## 5 Conclusion

The study concludes a pronounced incline in SCD‐related mortality since 2018, despite an overall decline since 1990. Significant disparities were noted, with females recording a higher increase in mortality, and steeper inclines in death across urban regions. Further, Hispanics noted the steepest rise in SCD, followed by Asian and Pacific Islanders and African Americans. Across regions of the US, the West has recorded the highest incline in mortality of all. Future research should attempt to characterize the risk factors accounting for these trends and assess global policies for affordable and equitable access of emergency healthcare services.

## Ethics Statement

The authors have nothing to report.

## Consent

The authors have nothing to report.

## Conflicts of Interest

The authors declare no conflicts of interest.

## Supporting information

Supplementary file.

## Data Availability

All data used in this study to formulate our results is publicly available online at the CDC WONDER database (https://wonder.cdc.gov/).

## References

[1] C. X. Wong , A. Brown , D. H. Lau , et al., “Epidemiology of Sudden Cardiac Death: Global and Regional Perspectives,” Heart, Lung and Circulation 28, no. 1 (2019): 6–14, 10.1016/J.HLC.2018.08.026. 30482683

[2] P. van der Bijl , T. Podlesnikar , J. J. Bax , and V. Delgado , “Predicción Del Riesgo De Muerte Súbita Cardiaca: El Papel De La Resonancia Magnética Cardiaca,” Revista Española de Cardiología 71, no. 11 (2018): 961–970, 10.1016/j.rec.2018.05.019. 29970349

[3] A. Kumar , D. M. Avishay , C. R. Jones , et al., “Sudden Cardiac Death: Epidemiology, Pathogenesis and Management,” Reviews in Cardiovascular Medicine 22, no. 1 (2021): 147–158, 10.31083/J.RCM.2021.01.207. 33792256

[4] C. S. Fox , J. C. Evans , M. G. Larson , W. B. Kannel , and D. Levy , “Temporal Trends in Coronary Heart Disease Mortality and Sudden Cardiac Death From 1950 to 1999,” Circulation 2004 Published online August 3, 10.1161/01.CIR.0000136993.34344.41. 15262842

[5] C. Amantea , E. Pilia , M. F. Rossi , et al., “Sudden Cardiac Death Among Workers: A Systematic Review and Meta‐Analysis,” Systematic Reviews 13, no. 1 (2024): 84, 10.1186/s13643-024-02504-5. 38461297 PMC10924409

[6] C. M. Albert , C. U. Chae , F. Grodstein , et al., “Prospective Study of Sudden Cardiac Death Among Women in the United States,” Circulation 107, no. 16 (2003): 2096–2101, 10.1161/01.CIR.0000065223.21530.11. 12695299

[7] R. D. Bagnall , R. G. Weintraub , J. Ingles , et al., “A Prospective Study of Sudden Cardiac Death Among Children and Young Adults,” New England Journal of Medicine 374, no. 25 (2016): 2441–2452, 10.1056/NEJMOA1510687. 27332903

[8] B. J. Petek , T. W. Churchill , N. Moulson , et al., “Sudden Cardiac Death in National Collegiate Athletic Association Athletes: A 20‐Year Study,” Circulation 149, no. 2 (2024): 80–90, 10.1161/CIRCULATIONAHA.123.065908/SUPPL_FILE/COTR149_02.PDF. 37955565 PMC10843024

[9] M. Zuin , S. Mohanty , R. Aggarwal , et al., “Trends in Sudden Cardiac Death Among Adults Aged 25 to 44 Years in the United States: An Analysis of 2 Large US Databases,” Journal of the American Heart Association 14, no. 1 (2025): e035722, 10.1161/JAHA.124.035722. 39692035 PMC12054444

[10] CDC WONDER. [cited 2025 Mar 11]. Available from: https://wonder.cdc.gov/.

[11] Multiple Cause of Death, 1999‐2020 Request. [cited 2025 Mar 11]. Available from: https://wonder.cdc.gov/mcd-icd10.html.

[12] ICD‐10 Version:2019. [cited 2025 Mar 11]. Available from: https://icd.who.int/browse10/2019/en.

[13] Joinpoint Regression Program. [cited 2025 Mar 11]. Available from: https://surveillance.cancer.gov/joinpoint/.

[14] P. Ponikowski , A. A. Voors , S. D. Anker , et al., Document Reviewers, “2016 ESC Guidelines for the Diagnosis and Treatment of Acute and Chronic Heart Failure: The Task Force for the Diagnosis and Treatment of Acute and Chronic Heart Failure of the European Society of Cardiology (ESC). Developed With the Special Contribution of the Heart Failure Association (HFA) of the ESC,” European Journal of Heart Failure 18, no. 8 (2016): 891–975, 10.1002/ejhf.592. 27207191

[15] S. He , H. Deng , J. Jiang , et al., “The Evolving Epidemiology of Elderly With Degenerative Valvular Heart Disease: The Guangzhou (China) Heart Study,” BioMed Research International 2021 (2021): 9982569, 10.1155/2021/9982569. 33981773 PMC8088353

[16] Centers for Disease Control and P. (C. D. C.) ., “State‐Specific Mortality From Sudden Cardiac Death—United States, 1999,” MMWR. Morbidity and Mortality Weekly Report 51, no. 6 (2002): 123–126. 11898927

[17] T. F. Platts‐Mills , B. Leacock , J. G. Cabañas , F. S. Shofer , and S. A. McLean , “Emergency Medical Services Use by the Elderly: Analysis of a Statewide Database,” Prehospital Emergency Care 14, no. 3 (2010): 329–333, 10.3109/10903127.2010.481759. 20507220

[18] Y. Ito , H. Sakaguchi , E. Tsuda , and K. Kurosaki , “Effect of Beta‐Blockers and Exercise Restriction on the Prevention of Sudden Cardiac Death in Pediatric Hypertrophic Cardiomyopathy,” Journal of Cardiology 83, no. 6 (2024): 407–414, 10.1016/j.jjcc.2023.11.009. 38043708

[19] B. Chong , J. Jayabaskaran , S. M. Jauhari , et al., “Global Burden of Cardiovascular Diseases: Projections From 2025 to 2050,” European Journal of Preventive Cardiology (2024): zwae281, Advance online publication. 10.1093/eurjpc/zwae281. 39270739

[20] N. H. Phelps , R. K. Singleton , B. Zhou , et al., NCD Risk Factor Collaboration (NCD‐RisC) ., “Worldwide Trends in Underweight and Obesity From 1990 to 2022: A Pooled Analysis of 3663 Population‐Representative Studies With 222 Million Children, Adolescents, and Adults,” The Lancet 403, no. 10431 (2024): 1027–1050, 10.1016/S0140-6736(23)02750-2. PMC7615769 38432237

[21] P. J. Hanson , F. Liu‐Fei , C. Ng , et al., “Characterization of COVID‐19‐associated Cardiac Injury: Evidence for a Multifactorial Disease in an Autopsy Cohort,” Laboratory Investigation 102, no. 8 (2022): 814–825, 10.1038/s41374-022-00783-x. 35437316 PMC9015288

[22] P. Sultanian , P. Lundgren , A. Strömsöe , et al., “Cardiac Arrest in COVID‐19: Characteristics and Outcomes of in‐ and Out‐of‐Hospital Cardiac Arrest. A Report From the Swedish Registry for Cardiopulmonary Resuscitation,” European Heart Journal 42, no. 11 (2021): 1094–1106, 10.1093/eurheartj/ehaa1067. 33543259 PMC7928992

[23] I. Hookana , M. A. E. Eskuri , L. Holmström , et al., “Age‐Related Trends of Ischemic Sudden Cardiac Death in Women,” International Journal of Cardiology 410 (2024): 132238, 10.1016/j.ijcard.2024.132238. 38838747

[24] P. Di Giosia , G. Passacquale , M. Petrarca , P. Giorgini , A. M. Marra , and A. Ferro , “Gender Differences in Cardiovascular Prophylaxis: Focus on Antiplatelet Treatment,” Pharmacological Research 119 (2017): 36–47, 10.1016/j.phrs.2017.01.025. 28131875

[25] K. A. Milner , M. Funk , S. Richards , R. M. Wilmes , V. Vaccarino , and H. M. Krumholz , “Gender Differences in Symptom Presentation Associated With Coronary Heart Disease,” American Journal of Cardiology 84, no. 4 (1999): 396–399, 10.1016/s0002-9149(99)00322-7. 10468075

[26] J. H. Lichtman , E. C. Leifheit‐Limson , E. Watanabe , et al., “Symptom Recognition and Healthcare Experiences of Young Women With Acute Myocardial Infarction,” Circulation. Cardiovascular Quality and Outcomes 8, no. 2 Suppl 1 (2015): 31–38, 10.1161/CIRCOUTCOMES.114.001612. PMC4801001 25714826

[27] C. M. Yong and W. F. Fearon , “Underrepresentation of Women in Revascularization Trials,” JAMA Cardiology 9, no. 6 (2024): 493–494, 10.1001/jamacardio.2024.0768. 38717765

[28] J. Tamargo , G. Rosano , T. Walther , et al., “Gender Differences in the Effects of Cardiovascular Drugs,” European Heart Journal—Cardiovascular Pharmacotherapy 3, no. 3 (2017): 163–182, 10.1093/ehjcvp/pvw042. 28329228

[29] M. Sherpa , “Are Women With Minimal to Mild Coronary Artery Disease at Higher Risk of Sudden Cardiac Death Than Men?” American Journal of Preventive Cardiology 19 (2024): 100826, 10.1016/j.ajpc.2024.100826.

[30] K. Reinier , J. Y. Moon , H. S. Chugh , et al., “Risk Factors for Sudden Cardiac Arrest Among Hispanic or Latino Adults In Southern California: Ventura PRESTO and HCHS/SOL,” Journal of the American Heart Association 12, no. 20 (2023): e030062, 10.1161/JAHA.123.030062. 37818701 PMC10757510

[31] N. Desai , C. M. Lora , J. P. Lash , and A. C. Ricardo , “CKD and ESRD in US Hispanics,” American Journal of Kidney Diseases 73, no. 1 (2019): 102–111, 10.1053/j.ajkd.2018.02.354. 29661541 PMC6186206

[32] G. Heiss , M. L. Snyder , Y. Teng , et al., “Prevalence of Metabolic Syndrome Among Hispanics/Latinos of Diverse Background: The Hispanic Community Health Study/Study of Latinos,” Diabetes Care 37, no. 8 (2014): 2391–2399, 10.2337/dc13-2505. 25061141 PMC4113166

[33] A. C. Rivera‐González , D. H. Roby , J. P. Stimpson , et al., “The Impact of Medicaid Funding Structures on Inequities in Health Care Access for Latinos in New York, Florida, and Puerto Rico,” Health Services Research 57, no. Suppl 2 (2022): 172–182, 10.1111/1475-6773.14036. PMC9660415 35861151

[34] S. Le Menestrel and G. Duncan National Academies of Sciences, Engineering, and Medicine; Division of Behavioral and Social Sciences and Education Committee on National Statistics; Board on Children, Youth, and Families; Committee on Building an Agenda to Reduce the Number of Children in Poverty by Half in 10 Years,” in A Roadmap to Reducing Child Poverty National Academies Press (US), 2019 Feb 28. 2, A Demographic Portrait of Child Poverty in the United States. 31593382

[35] B. Waitzfelder , L. Palaniappan , A. Varga , et al., “Prevalence of Cardiovascular Disease Among Asian, Pacific Islander and Multi‐Race Populations in Hawai'I and California,” BMC Public Health 23, no. 1 (2023): 885, 10.1186/s12889-023-15795-5. 37189145 PMC10184427

[36] N. S. Shah , G. M. Giase , L. C. Petito , et al., “Outcomes in Patients Hospitalized for COVID‐19 Among Asian, Pacific Islander, and Hispanic Subgroups in the American Heart Association COVID‐19 Registry,” American Journal of Medicine Open 1 (2021): 100003, 10.1016/j.ajmo.2021.100003. 34918003 PMC8606255

[37] F. R. Giugni , A. N. Duarte‐Neto , L. F. F. da Silva , et al., “Younger Age Is Associated With Cardiovascular Pathological Phenotype of Severe COVID‐19 at Autopsy,” Frontiers in Medicine 10 (2024): 1327415, 10.3389/fmed.2023.1327415. 38259848 PMC10801169

[38] L. Guo , S. Torii , R. Fernandez , et al., “Genetic Variants Associated With Unexplained Sudden Cardiac Death in Adult White and African American Individuals,” JAMA Cardiology 6, no. 9 (2021): 1013–1022, 10.1001/jamacardio.2021.1573. 34076677 PMC8173469

[39] R. Deo , M. M. Safford , Y. A. Khodneva , et al., “Differences in Risk of Sudden Cardiac Death Between Blacks and Whites,” Journal of the American College of Cardiology 72, no. 20 (2018): 2431–2439, 10.1016/j.jacc.2018.08.2173. 30442286 PMC9704756

[40] J. F. Meschia , “Pacemakers as Atrial Fibrillation Detectors: Finding Racial Differences and Opportunities for Preventing Stroke,” Journal of the American Heart Association 5, no. 2 (2016): e003090, 10.1161/JAHA.115.003090. 26873690 PMC4802437

[41] C. L. Colvin , J. B. King , S. Oparil , et al., “Association of Race/Ethnicity‐Specific Changes in Antihypertensive Medication Classes Initiated Among Medicare Beneficiaries With the Eighth Joint National Committee Panel Member Report,” JAMA Network Open 3, no. 11 (2020): e2025127, 10.1001/jamanetworkopen.2020.25127. 33206191 PMC7675104

[42] L. A. Eberly , K. Shultz , M. Merino , et al., “Cardiovascular Disease Burden and Outcomes Among American Indian and Alaska Native Medicare Beneficiaries,” JAMA Network Open 6, no. 9 (2023): e2334923, 10.1001/jamanetworkopen.2023.34923. 37738051 PMC10517375

[43] M. A. Jim , E. Arias , D. S. Seneca , et al., “Racial Misclassification of American Indians and Alaska Natives by Indian Health Service Contract Health Service Delivery Area,” American Journal of Public Health 104, no. Suppl 3 (2014): S295–S302, 10.2105/AJPH.2014.301933. 24754617 PMC4035863

[44] A. Jana , “Exposure to Urban Environment and Associated Lifestyle Diseases Among Older Adults in India: Evidence From LASI,” Cities & Health 9, no. 1 (2025): 15–29, 10.1080/23748834.2024.2439644.

[45] A. Svensson , B. Nilsson , E. Lantz , A. Bremer , K. Årestedt , and J. Israelsson , “Response Times in Rural Areas for Emergency Medical Services, Fire and Rescue Services and Voluntary First Responders During Out‐Of‐Hospital Cardiac Arrests,” Resuscitation Plus 17 (2024): 100548, 10.1016/j.resplu.2023.100548. 38292470 PMC10825318

[46] L. Wang , J. Liu , P. Yin , et al., “Mortality Risk and Burden of Sudden Cardiac Arrest Associated With Hot Nights, Heatwaves, Cold Spells, and Non‐Optimum Temperatures in 0.88 Million Patients: An Individual‐Level Case‐Crossover Study,” Science of the Total Environment 949 (2024): 175208, 10.1016/j.scitotenv.2024.175208. 39097015

[47] A. Bunker , J. Wildenhain , A. Vandenbergh , et al., “Effects of Air Temperature on Climate‐Sensitive Mortality and Morbidity Outcomes in the Elderly; a Systematic Review and Meta‐Analysis of Epidemiological Evidence,” EBioMedicine 6 (2016): 258–268, 10.1016/j.ebiom.2016.02.034. 27211569 PMC4856745

[48] V. Parcha , R. Kalra , S. S. Suri , et al., “Geographic Variation in Cardiovascular Health Among American Adults,” Mayo Clinic Proceedings 96, no. 7 (2021): 1770–1781, 10.1016/j.mayocp.2020.12.034. 33775420 PMC8260439

[49] J. T. Kullgren , C. G. McLaughlin , N. Mitra , and K. Armstrong , “Nonfinancial Barriers and Access to Care for U.S. Adults,” Health Services Research 47, no. 1 pt. 2 (2012): 462–485, 10.1111/j.1475-6773.2011.01308.x. 22092449 PMC3393009

[50] S. L. McComish , X. Liu , F. T. Martinez , J. Y. Zhou , and S. Y. Tolmachev , “Misclassification of Causes of Death Among a Small All‐Autopsied Group of Former Nuclear Workers: Death Certificates vs. Autopsy Reports,” PLoS One 19, no. 5 (2024): e0302069, 10.1371/journal.pone.0302069. 38701098 PMC11068187

