# Supplementary material for: Trends in Sudden Cardiac Death Related Mortality in Adults in the United States: A CDC WONDER Database Analysis, 1999–2020
Source: Clin Cardiol. 2025 Jul 15;48(7):e70180. doi: 10.1002/clc.70180 (PMC12261031; doi:10.1002/clc.70180)

**Supplementary Table 1:** Age-Adjusted Mortality Rates (AAMR) from Sudden Cardiac Deaths in the US by Gender (1999-2020).

| **Sex** | **Year** | **Age Adjusted Rate** | **Age Adjusted Rate Lower 95% Confidence Interval** | **Age Adjusted Rate Upper 95% Confidence Interval** |
| --- | --- | --- | --- | --- |
| Female | 1999 | 5.0313 | 4.8966 | 5.1661 |
| Female | 2000 | 5.0294 | 4.8952 | 5.1637 |
| Female | 2001 | 4.8321 | 4.7014 | 4.9628 |
| Female | 2002 | 4.9389 | 4.8073 | 5.0705 |
| Female | 2003 | 4.7325 | 4.6046 | 4.8603 |
| Female | 2004 | 4.5679 | 4.4423 | 4.6935 |
| Female | 2005 | 4.6268 | 4.5018 | 4.7518 |
| Female | 2006 | 4.644 | 4.5194 | 4.7686 |
| Female | 2007 | 4.3762 | 4.2556 | 4.4968 |
| Female | 2008 | 4.4454 | 4.3251 | 4.5657 |
| Female | 2009 | 4.3285 | 4.2112 | 4.4458 |
| Female | 2010 | 4.2609 | 4.1446 | 4.3773 |
| Female | 2011 | 4.0921 | 3.9788 | 4.2053 |
| Female | 2012 | 4.003 | 3.8924 | 4.1136 |
| Female | 2013 | 3.8344 | 3.7266 | 3.9422 |
| Female | 2014 | 3.7628 | 3.6566 | 3.869 |
| Female | 2015 | 3.7434 | 3.638 | 3.8488 |
| Female | 2016 | 3.4046 | 3.3058 | 3.5035 |
| Female | 2017 | 3.2938 | 3.1975 | 3.3901 |
| Female | 2018 | 3.3462 | 3.2493 | 3.4431 |
| Female | 2019 | 3.3058 | 3.2107 | 3.4009 |
| Female | 2020 | 3.852 | 3.7493 | 3.9548 |
| Male | 1999 | 9.5022 | 9.2733 | 9.731 |
| Male | 2000 | 9.5892 | 9.3616 | 9.8168 |
| Male | 2001 | 9.2432 | 9.0221 | 9.4642 |
| Male | 2002 | 9.3233 | 9.1035 | 9.5432 |
| Male | 2003 | 9.232 | 9.0155 | 9.4485 |
| Male | 2004 | 8.6199 | 8.414 | 8.8257 |
| Male | 2005 | 8.8529 | 8.6465 | 9.0593 |
| Male | 2006 | 8.6206 | 8.4202 | 8.821 |
| Male | 2007 | 8.6687 | 8.4699 | 8.8674 |
| Male | 2008 | 8.2977 | 8.1056 | 8.4897 |
| Male | 2009 | 8.1952 | 8.0064 | 8.3839 |
| Male | 2010 | 7.9503 | 7.7661 | 8.1344 |
| Male | 2011 | 7.7544 | 7.5751 | 7.9337 |
| Male | 2012 | 7.495 | 7.3218 | 7.6682 |
| Male | 2013 | 7.556 | 7.3841 | 7.7278 |
| Male | 2014 | 7.2396 | 7.0737 | 7.4056 |
| Male | 2015 | 7.1394 | 6.9768 | 7.302 |
| Male | 2016 | 6.8908 | 6.7322 | 7.0494 |
| Male | 2017 | 6.7258 | 6.5713 | 6.8802 |
| Male | 2018 | 6.6743 | 6.5225 | 6.8262 |
| Male | 2019 | 6.6503 | 6.5004 | 6.8003 |
| Male | 2020 | 7.6293 | 7.4699 | 7.7887 |

**Supplementary Figure 1:** Age-Adjusted Mortality Rates Annual Percentage Change (APC) from Sudden Cardiac Deaths in the US by Gender (1999-2020).


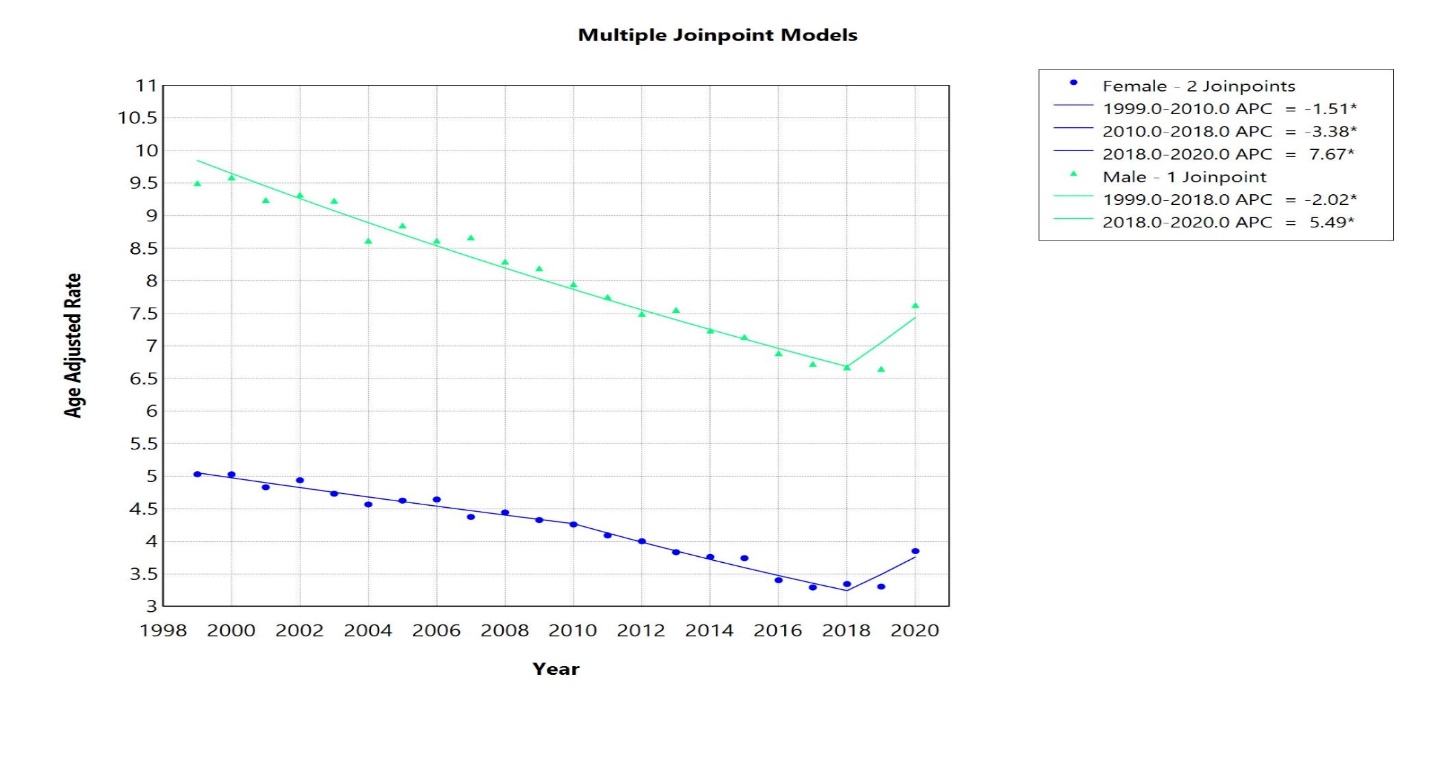


**Supplementary Table 2:** Age-Adjusted Mortality Rates (AAMR) from Sudden Cardiac Deaths in the US by Race (1999-2020).

| **Race** | **Year** | **Age Adjusted Rate** | **Age Adjusted Rate Lower 95% Confidence Interval** | **Age Adjusted Rate Upper 95% Confidence Interval** |
| --- | --- | --- | --- | --- |
| American Indian or Alaska Native | 1999 | 7.163 | 5.3497 | 9.3933 |
| American Indian or Alaska Native | 2000 | 7.4244 | 5.6232 | 9.6192 |
| American Indian or Alaska Native | 2001 | 5.2281 | 3.7518 | 7.0925 |
| American Indian or Alaska Native | 2002 | 4.5368 | 3.2261 | 6.2019 |
| American Indian or Alaska Native | 2003 | 5.5832 | 4.144 | 7.3608 |
| American Indian or Alaska Native | 2004 | 6.1399 | 4.6254 | 7.9919 |
| American Indian or Alaska Native | 2005 | 5.581 | 4.2485 | 7.1991 |
| American Indian or Alaska Native | 2006 | 4.0627 | 3.0056 | 5.3711 |
| American Indian or Alaska Native | 2007 | 5.4686 | 4.2022 | 6.9967 |
| American Indian or Alaska Native | 2008 | 4.3447 | 3.2639 | 5.6689 |
| American Indian or Alaska Native | 2009 | 4.2844 | 3.2276 | 5.5767 |
| American Indian or Alaska Native | 2010 | 2.744 | 1.932 | 3.7823 |
| American Indian or Alaska Native | 2011 | 3.9462 | 3.0041 | 5.0904 |
| American Indian or Alaska Native | 2012 | 3.9779 | 3.0568 | 5.0895 |
| American Indian or Alaska Native | 2013 | 3.3289 | 2.5078 | 4.333 |
| American Indian or Alaska Native | 2014 | 3.0711 | 2.3136 | 3.9975 |
| American Indian or Alaska Native | 2015 | 3.4228 | 2.658 | 4.3393 |
| American Indian or Alaska Native | 2016 | 3.2669 | 2.4989 | 4.1965 |
| American Indian or Alaska Native | 2017 | 3.4388 | 2.6704 | 4.3595 |
| American Indian or Alaska Native | 2018 | 3.302 | 2.5928 | 4.1453 |
| American Indian or Alaska Native | 2019 | 3.0536 | 2.3665 | 3.8779 |
| American Indian or Alaska Native | 2020 | 3.4174 | 2.7297 | 4.2257 |
| Asian or Pacific Islander | 1999 | 2.2234 | 1.7735 | 2.7528 |
| Asian or Pacific Islander | 2000 | 2.0918 | 1.6777 | 2.5771 |
| Asian or Pacific Islander | 2001 | 2.1369 | 1.6964 | 2.5775 |
| Asian or Pacific Islander | 2002 | 1.9308 | 1.5387 | 2.323 |
| Asian or Pacific Islander | 2003 | 2.0303 | 1.6345 | 2.4262 |
| Asian or Pacific Islander | 2004 | 2.1202 | 1.7336 | 2.5068 |
| Asian or Pacific Islander | 2005 | 1.8697 | 1.521 | 2.2184 |
| Asian or Pacific Islander | 2006 | 2.1474 | 1.7819 | 2.5129 |
| Asian or Pacific Islander | 2007 | 2.0202 | 1.6736 | 2.3669 |
| Asian or Pacific Islander | 2008 | 1.8888 | 1.5566 | 2.2209 |
| Asian or Pacific Islander | 2009 | 2.1129 | 1.7815 | 2.4442 |
| Asian or Pacific Islander | 2010 | 1.6848 | 1.4004 | 1.9692 |
| Asian or Pacific Islander | 2011 | 1.7208 | 1.4389 | 2.0028 |
| Asian or Pacific Islander | 2012 | 1.8426 | 1.5605 | 2.1247 |
| Asian or Pacific Islander | 2013 | 1.6442 | 1.3876 | 1.9009 |
| Asian or Pacific Islander | 2014 | 1.7002 | 1.4497 | 1.9507 |
| Asian or Pacific Islander | 2015 | 1.7039 | 1.4586 | 1.9492 |
| Asian or Pacific Islander | 2016 | 1.7865 | 1.544 | 2.029 |
| Asian or Pacific Islander | 2017 | 1.4549 | 1.2411 | 1.6686 |
| Asian or Pacific Islander | 2018 | 1.4189 | 1.2123 | 1.6255 |
| Asian or Pacific Islander | 2019 | 1.5128 | 1.305 | 1.7205 |
| Asian or Pacific Islander | 2020 | 2.026 | 1.7908 | 2.2613 |
| Black or African American | 1999 | 8.7162 | 8.2487 | 9.1837 |
| Black or African American | 2000 | 9.4742 | 8.9906 | 9.9578 |
| Black or African American | 2001 | 8.9875 | 8.5205 | 9.4545 |
| Black or African American | 2002 | 10.0734 | 9.5827 | 10.5641 |
| Black or African American | 2003 | 10.1494 | 9.6623 | 10.6364 |
| Black or African American | 2004 | 9.1704 | 8.7153 | 9.6254 |
| Black or African American | 2005 | 10.0387 | 9.5696 | 10.5079 |
| Black or African American | 2006 | 10.5654 | 10.086 | 11.0448 |
| Black or African American | 2007 | 9.7575 | 9.3028 | 10.2122 |
| Black or African American | 2008 | 10.0155 | 9.5615 | 10.4696 |
| Black or African American | 2009 | 9.2045 | 8.7736 | 9.6355 |
| Black or African American | 2010 | 9.253 | 8.8249 | 9.6812 |
| Black or African American | 2011 | 8.4137 | 8.0132 | 8.8141 |
| Black or African American | 2012 | 7.5211 | 7.1501 | 7.892 |
| Black or African American | 2013 | 7.8806 | 7.5089 | 8.2522 |
| Black or African American | 2014 | 7.4619 | 7.1056 | 7.8183 |
| Black or African American | 2015 | 7.1284 | 6.7871 | 7.4697 |
| Black or African American | 2016 | 6.9516 | 6.6185 | 7.2846 |
| Black or African American | 2017 | 6.2605 | 5.95 | 6.5711 |
| Black or African American | 2018 | 6.1713 | 5.867 | 6.4755 |
| Black or African American | 2019 | 6.1213 | 5.8234 | 6.4191 |
| Black or African American | 2020 | 7.5943 | 7.2675 | 7.9211 |
| White | 1999 | 6.8944 | 6.7641 | 7.0246 |
| White | 2000 | 6.8691 | 6.7399 | 6.9983 |
| White | 2001 | 6.681 | 6.5542 | 6.8078 |
| White | 2002 | 6.6731 | 6.5473 | 6.7988 |
| White | 2003 | 6.4749 | 6.3519 | 6.5979 |
| White | 2004 | 6.1896 | 6.0699 | 6.3093 |
| White | 2005 | 6.2673 | 6.1479 | 6.3867 |
| White | 2006 | 6.1305 | 6.0136 | 6.2475 |
| White | 2007 | 6.0423 | 5.927 | 6.1576 |
| White | 2008 | 5.9063 | 5.7933 | 6.0194 |
| White | 2009 | 5.8495 | 5.7382 | 5.9608 |
| White | 2010 | 5.7425 | 5.6323 | 5.8527 |
| White | 2011 | 5.6355 | 5.5271 | 5.744 |
| White | 2012 | 5.5674 | 5.4607 | 5.6741 |
| White | 2013 | 5.4068 | 5.3031 | 5.5106 |
| White | 2014 | 5.2915 | 5.1891 | 5.3938 |
| White | 2015 | 5.2556 | 5.1545 | 5.3567 |
| White | 2016 | 4.9623 | 4.8644 | 5.0602 |
| White | 2017 | 4.9117 | 4.8154 | 5.0079 |
| White | 2018 | 4.9145 | 4.8189 | 5.01 |
| White | 2019 | 4.8954 | 4.8008 | 4.99 |
| White | 2020 | 5.5526 | 5.4522 | 5.653 |
| Hispanic or Latino | 1999 | 3.1031 | 2.7157 | 3.4905 |
| Hispanic or Latino | 2000 | 2.9583 | 2.5936 | 3.323 |
| Hispanic or Latino | 2001 | 2.9721 | 2.6137 | 3.3305 |
| Hispanic or Latino | 2002 | 2.5821 | 2.2586 | 2.9055 |
| Hispanic or Latino | 2003 | 2.5056 | 2.1929 | 2.8182 |
| Hispanic or Latino | 2004 | 2.3657 | 2.0712 | 2.6602 |
| Hispanic or Latino | 2005 | 2.7626 | 2.4536 | 3.0716 |
| Hispanic or Latino | 2006 | 2.388 | 2.1112 | 2.6648 |
| Hispanic or Latino | 2007 | 2.3925 | 2.126 | 2.659 |
| Hispanic or Latino | 2008 | 2.4772 | 2.2081 | 2.7463 |
| Hispanic or Latino | 2009 | 2.6108 | 2.3415 | 2.8802 |
| Hispanic or Latino | 2010 | 2.2072 | 1.9643 | 2.4501 |
| Hispanic or Latino | 2011 | 1.9971 | 1.7771 | 2.2172 |
| Hispanic or Latino | 2012 | 2.2023 | 1.9783 | 2.4262 |
| Hispanic or Latino | 2013 | 2.1541 | 1.9396 | 2.3686 |
| Hispanic or Latino | 2014 | 2.0671 | 1.8654 | 2.2688 |
| Hispanic or Latino | 2015 | 2.2303 | 2.026 | 2.4346 |
| Hispanic or Latino | 2016 | 1.892 | 1.7084 | 2.0757 |
| Hispanic or Latino | 2017 | 1.6392 | 1.4746 | 1.8037 |
| Hispanic or Latino | 2018 | 1.931 | 1.755 | 2.107 |
| Hispanic or Latino | 2019 | 1.8415 | 1.6729 | 2.0101 |
| Hispanic or Latino | 2020 | 2.4459 | 2.2565 | 2.6352 |

**Supplementary Figure 2:** Age-Adjusted Mortality Rates Annual Percentage Change (APC) from Sudden Cardiac Deaths in the US by Race (1999-2020).


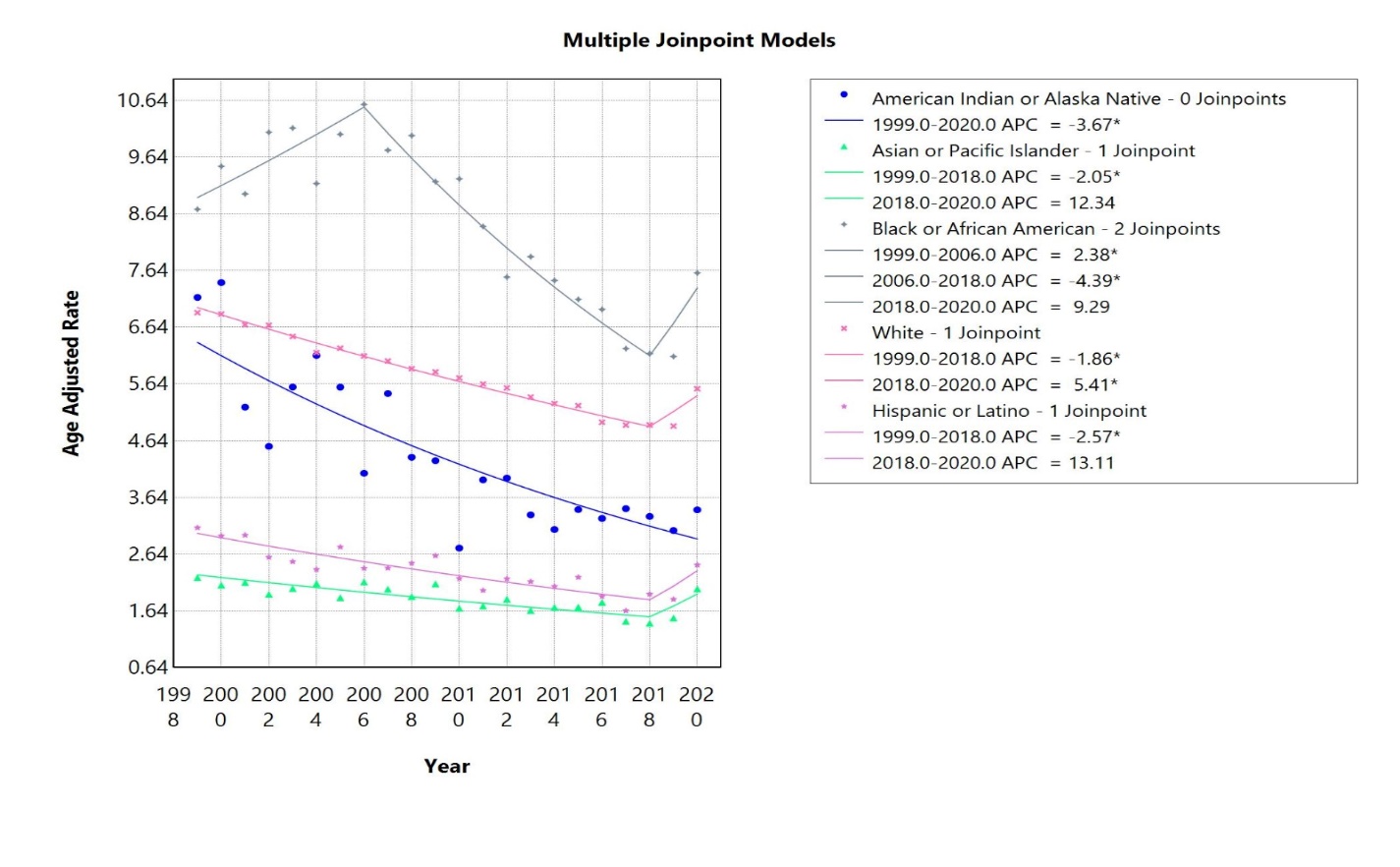


**Supplementary Table 3:** Age-Adjusted Mortality Rates (AAMR) from Sudden Cardiac Deaths in the US by Metropolitan Status (1999-2020).

| **2013 Urbanization** | **Year** | **Age Adjusted Rate** | **Age Adjusted Rate Lower 95% Confidence Interval** | **Age Adjusted Rate Upper 95% Confidence Interval** |
| --- | --- | --- | --- | --- |
| Metropolitan | 1999 | 5.8286 | 5.7037 | 5.9535 |
| Metropolitan | 2000 | 5.8209 | 5.6967 | 5.9451 |
| Metropolitan | 2001 | 5.6221 | 5.5012 | 5.743 |
| Metropolitan | 2002 | 5.6687 | 5.5483 | 5.789 |
| Metropolitan | 2003 | 5.5959 | 5.4774 | 5.7144 |
| Metropolitan | 2004 | 5.2154 | 5.1022 | 5.3287 |
| Metropolitan | 2005 | 5.2283 | 5.1161 | 5.3405 |
| Metropolitan | 2006 | 5.1325 | 5.0225 | 5.2426 |
| Metropolitan | 2007 | 5.0948 | 4.9862 | 5.2034 |
| Metropolitan | 2008 | 4.9992 | 4.8926 | 5.1059 |
| Metropolitan | 2009 | 4.9414 | 4.8366 | 5.0462 |
| Metropolitan | 2010 | 4.7976 | 4.6949 | 4.9002 |
| Metropolitan | 2011 | 4.6608 | 4.5608 | 4.7608 |
| Metropolitan | 2012 | 4.5998 | 4.5019 | 4.6977 |
| Metropolitan | 2013 | 4.4992 | 4.4034 | 4.5949 |
| Metropolitan | 2014 | 4.3058 | 4.213 | 4.3987 |
| Metropolitan | 2015 | 4.2852 | 4.1935 | 4.3769 |
| Metropolitan | 2016 | 3.9871 | 3.8996 | 4.0746 |
| Metropolitan | 2017 | 3.7887 | 3.7042 | 3.8732 |
| Metropolitan | 2018 | 3.8722 | 3.7879 | 3.9564 |
| Metropolitan | 2019 | 3.8635 | 3.7798 | 3.9473 |
| Metropolitan | 2020 | 4.4799 | 4.3905 | 4.5693 |
| Non-Metropolitan | 1999 | 11.8875 | 11.5152 | 12.2598 |
| Non-Metropolitan | 2000 | 12.3458 | 11.9672 | 12.7244 |
| Non-Metropolitan | 2001 | 11.8615 | 11.4923 | 12.2308 |
| Non-Metropolitan | 2002 | 12.1943 | 11.8207 | 12.5679 |
| Non-Metropolitan | 2003 | 11.7247 | 11.36 | 12.0894 |
| Non-Metropolitan | 2004 | 11.5124 | 11.1525 | 11.8722 |
| Non-Metropolitan | 2005 | 12.3229 | 11.9523 | 12.6935 |
| Non-Metropolitan | 2006 | 12.3868 | 12.018 | 12.7557 |
| Non-Metropolitan | 2007 | 11.6733 | 11.3179 | 12.0287 |
| Non-Metropolitan | 2008 | 11.7168 | 11.3619 | 12.0717 |
| Non-Metropolitan | 2009 | 11.315 | 10.969 | 11.661 |
| Non-Metropolitan | 2010 | 11.1932 | 10.8506 | 11.5359 |
| Non-Metropolitan | 2011 | 10.9419 | 10.603 | 11.2809 |
| Non-Metropolitan | 2012 | 10.3888 | 10.0615 | 10.7161 |
| Non-Metropolitan | 2013 | 10.4733 | 10.1452 | 10.8013 |
| Non-Metropolitan | 2014 | 10.5855 | 10.2559 | 10.915 |
| Non-Metropolitan | 2015 | 10.4557 | 10.1281 | 10.7832 |
| Non-Metropolitan | 2016 | 10.3024 | 9.9771 | 10.6277 |
| Non-Metropolitan | 2017 | 10.498 | 10.1751 | 10.8209 |
| Non-Metropolitan | 2018 | 10.0714 | 9.7549 | 10.388 |
| Non-Metropolitan | 2019 | 10.094 | 9.7786 | 10.4094 |
| Non-Metropolitan | 2020 | 11.6148 | 11.2756 | 11.954 |

**Supplementary Figure 3:** Age-Adjusted Mortality Rates Annual Percentage Change (APC) from Sudden Cardiac Deaths in the US by Metropolitan Status (1999-2020).


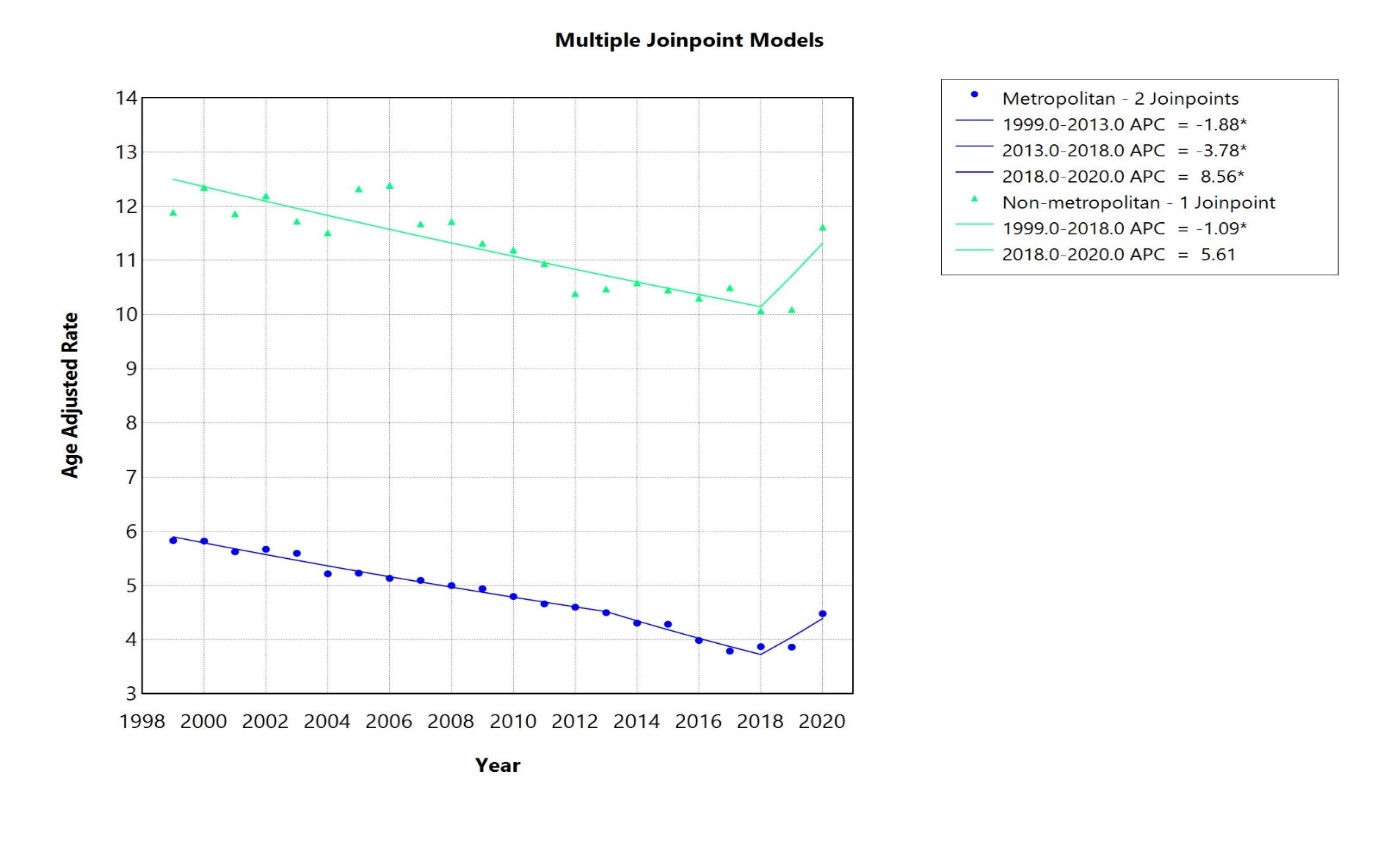


**Supplementary Table 4:** Age-Adjusted Mortality Rates (AAMR) from Sudden Cardiac Deaths in the US by Region (1999-2020).

| Census Region | Year | Age Adjusted Rate | Age Adjusted Rate Lower 95% Confidence Interval | Age Adjusted Rate Upper 95% Confidence Interval |
| --- | --- | --- | --- | --- |
| Census Region 1: Northeast | 1999 | 5.7737 | 5.5294 | 6.018 |
| Census Region 1: Northeast | 2000 | 5.7544 | 5.5114 | 5.9973 |
| Census Region 1: Northeast | 2001 | 5.4381 | 5.2034 | 5.6728 |
| Census Region 1: Northeast | 2002 | 5.8516 | 5.6099 | 6.0932 |
| Census Region 1: Northeast | 2003 | 5.4777 | 5.2449 | 5.7105 |
| Census Region 1: Northeast | 2004 | 5.1984 | 4.9722 | 5.4246 |
| Census Region 1: Northeast | 2005 | 4.9807 | 4.7602 | 5.2013 |
| Census Region 1: Northeast | 2006 | 4.7606 | 4.5469 | 4.9743 |
| Census Region 1: Northeast | 2007 | 4.7028 | 4.4914 | 4.9142 |
| Census Region 1: Northeast | 2008 | 4.579 | 4.3714 | 4.7866 |
| Census Region 1: Northeast | 2009 | 4.846 | 4.6329 | 5.0591 |
| Census Region 1: Northeast | 2010 | 4.7953 | 4.5842 | 5.0064 |
| Census Region 1: Northeast | 2011 | 5.0045 | 4.79 | 5.219 |
| Census Region 1: Northeast | 2012 | 4.7282 | 4.5217 | 4.9347 |
| Census Region 1: Northeast | 2013 | 4.5878 | 4.3868 | 4.7888 |
| Census Region 1: Northeast | 2014 | 4.2703 | 4.0768 | 4.4638 |
| Census Region 1: Northeast | 2015 | 4.3182 | 4.124 | 4.5124 |
| Census Region 1: Northeast | 2016 | 3.8848 | 3.7021 | 4.0675 |
| Census Region 1: Northeast | 2017 | 4.1894 | 4.0012 | 4.3776 |
| Census Region 1: Northeast | 2018 | 4.2645 | 4.0755 | 4.4536 |
| Census Region 1: Northeast | 2019 | 4.2924 | 4.1041 | 4.4806 |
| Census Region 1: Northeast | 2020 | 4.7143 | 4.5179 | 4.9108 |
| Census Region 2: Midwest | 1999 | 7.9639 | 7.6943 | 8.2334 |
| Census Region 2: Midwest | 2000 | 8.1213 | 7.8501 | 8.3924 |
| Census Region 2: Midwest | 2001 | 8.0442 | 7.7763 | 8.3122 |
| Census Region 2: Midwest | 2002 | 7.5197 | 7.2621 | 7.7773 |
| Census Region 2: Midwest | 2003 | 7.3885 | 7.1342 | 7.6428 |
| Census Region 2: Midwest | 2004 | 7.0875 | 6.84 | 7.335 |
| Census Region 2: Midwest | 2005 | 6.7643 | 6.5247 | 7.0039 |
| Census Region 2: Midwest | 2006 | 6.7297 | 6.4918 | 6.9677 |
| Census Region 2: Midwest | 2007 | 6.4628 | 6.2315 | 6.6942 |
| Census Region 2: Midwest | 2008 | 6.4608 | 6.2309 | 6.6907 |
| Census Region 2: Midwest | 2009 | 6.1506 | 5.9276 | 6.3735 |
| Census Region 2: Midwest | 2010 | 6.0578 | 5.837 | 6.2787 |
| Census Region 2: Midwest | 2011 | 5.9934 | 5.7756 | 6.2112 |
| Census Region 2: Midwest | 2012 | 6.2026 | 5.9831 | 6.4221 |
| Census Region 2: Midwest | 2013 | 6.2593 | 6.0398 | 6.4788 |
| Census Region 2: Midwest | 2014 | 6.4361 | 6.2154 | 6.6568 |
| Census Region 2: Midwest | 2015 | 6.4891 | 6.2678 | 6.7104 |
| Census Region 2: Midwest | 2016 | 6.1921 | 5.9771 | 6.4072 |
| Census Region 2: Midwest | 2017 | 5.9931 | 5.7847 | 6.2016 |
| Census Region 2: Midwest | 2018 | 5.7159 | 5.513 | 5.9189 |
| Census Region 2: Midwest | 2019 | 5.5862 | 5.3883 | 5.784 |
| Census Region 2: Midwest | 2020 | 6.2298 | 6.0199 | 6.4397 |
| Census Region 3: South | 1999 | 8.6528 | 8.4212 | 8.8845 |
| Census Region 3: South | 2000 | 8.9278 | 8.6944 | 9.1612 |
| Census Region 3: South | 2001 | 8.5338 | 8.3073 | 8.7602 |
| Census Region 3: South | 2002 | 8.869 | 8.6402 | 9.0978 |
| Census Region 3: South | 2003 | 8.692 | 8.4676 | 8.9163 |
| Census Region 3: South | 2004 | 8.1291 | 7.9144 | 8.3438 |
| Census Region 3: South | 2005 | 8.7347 | 8.5143 | 8.9551 |
| Census Region 3: South | 2006 | 8.7531 | 8.5351 | 8.9712 |
| Census Region 3: South | 2007 | 8.6895 | 8.4741 | 8.9049 |
| Census Region 3: South | 2008 | 8.3881 | 8.1794 | 8.5969 |
| Census Region 3: South | 2009 | 8.0389 | 7.8374 | 8.2405 |
| Census Region 3: South | 2010 | 7.9572 | 7.7577 | 8.1567 |
| Census Region 3: South | 2011 | 7.3654 | 7.1762 | 7.5546 |
| Census Region 3: South | 2012 | 7.0127 | 6.8301 | 7.1953 |
| Census Region 3: South | 2013 | 6.8215 | 6.6443 | 6.9987 |
| Census Region 3: South | 2014 | 6.3325 | 6.1634 | 6.5017 |
| Census Region 3: South | 2015 | 6.1934 | 6.0281 | 6.3588 |
| Census Region 3: South | 2016 | 5.9065 | 5.7464 | 6.0666 |
| Census Region 3: South | 2017 | 5.7203 | 5.565 | 5.8756 |
| Census Region 3: South | 2018 | 5.8632 | 5.7084 | 6.018 |
| Census Region 3: South | 2019 | 5.8265 | 5.6732 | 5.9797 |
| Census Region 3: South | 2020 | 6.7307 | 6.5676 | 6.8938 |
| Census Region 4: West | 1999 | 4.0626 | 3.8519 | 4.2733 |
| Census Region 4: West | 2000 | 3.6519 | 3.4541 | 3.8497 |
| Census Region 4: West | 2001 | 3.4929 | 3.3023 | 3.6835 |
| Census Region 4: West | 2002 | 3.5313 | 3.3417 | 3.721 |
| Census Region 4: West | 2003 | 3.5965 | 3.4081 | 3.7849 |
| Census Region 4: West | 2004 | 3.5416 | 3.3559 | 3.7273 |
| Census Region 4: West | 2005 | 3.8216 | 3.6312 | 4.0121 |
| Census Region 4: West | 2006 | 3.6523 | 3.4691 | 3.8356 |
| Census Region 4: West | 2007 | 3.4244 | 3.2487 | 3.6002 |
| Census Region 4: West | 2008 | 3.496 | 3.3208 | 3.6713 |
| Census Region 4: West | 2009 | 3.661 | 3.4842 | 3.8378 |
| Census Region 4: West | 2010 | 3.2947 | 3.1294 | 3.46 |
| Census Region 4: West | 2011 | 3.326 | 3.1611 | 3.4908 |
| Census Region 4: West | 2012 | 3.3366 | 3.1747 | 3.4984 |
| Census Region 4: West | 2013 | 3.2976 | 3.1384 | 3.4568 |
| Census Region 4: West | 2014 | 3.4324 | 3.2727 | 3.5921 |
| Census Region 4: West | 2015 | 3.445 | 3.285 | 3.6049 |
| Census Region 4: West | 2016 | 3.2949 | 3.1403 | 3.4495 |
| Census Region 4: West | 2017 | 2.8967 | 2.7539 | 3.0394 |
| Census Region 4: West | 2018 | 2.8468 | 2.7071 | 2.9865 |
| Census Region 4: West | 2019 | 2.8941 | 2.755 | 3.0333 |
| Census Region 4: West | 2020 | 3.6948 | 3.5391 | 3.8506 |

**Supplementary Figure 4:** Age-Adjusted Mortality Rates Annual Percentage Change (APC) from Sudden Cardiac Deaths in the US by Region (1999-2020).


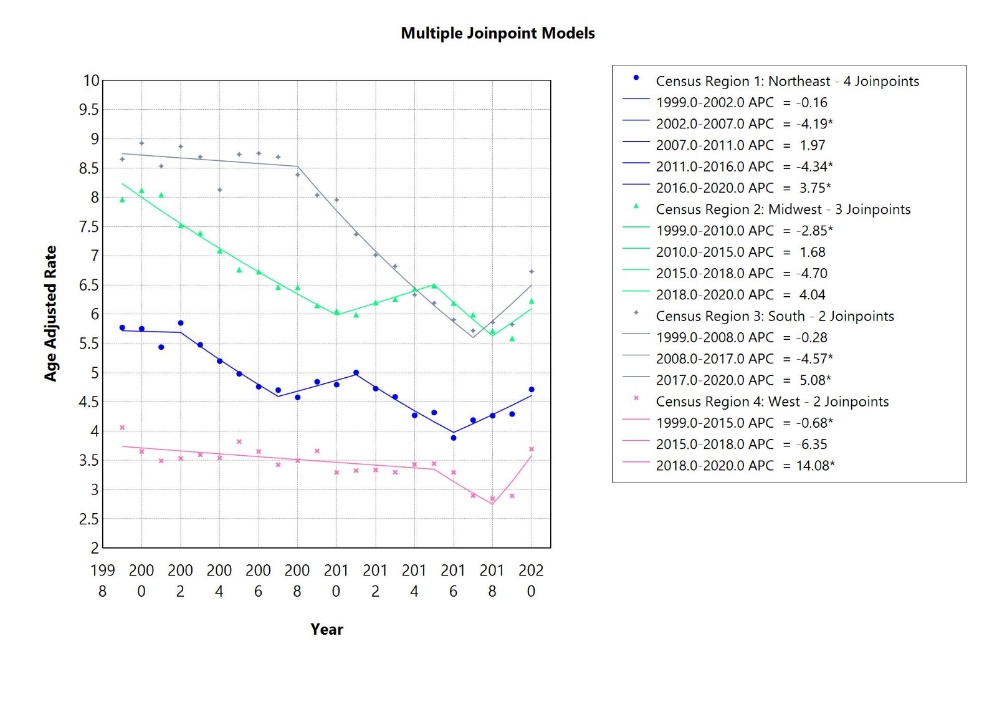


**Supplementary figure 5:** Age-Adjusted Mortality Rates Annual Percentage Change (APC) from Sudden Cardiac Deaths in the US Annual Trends (1999-2020)


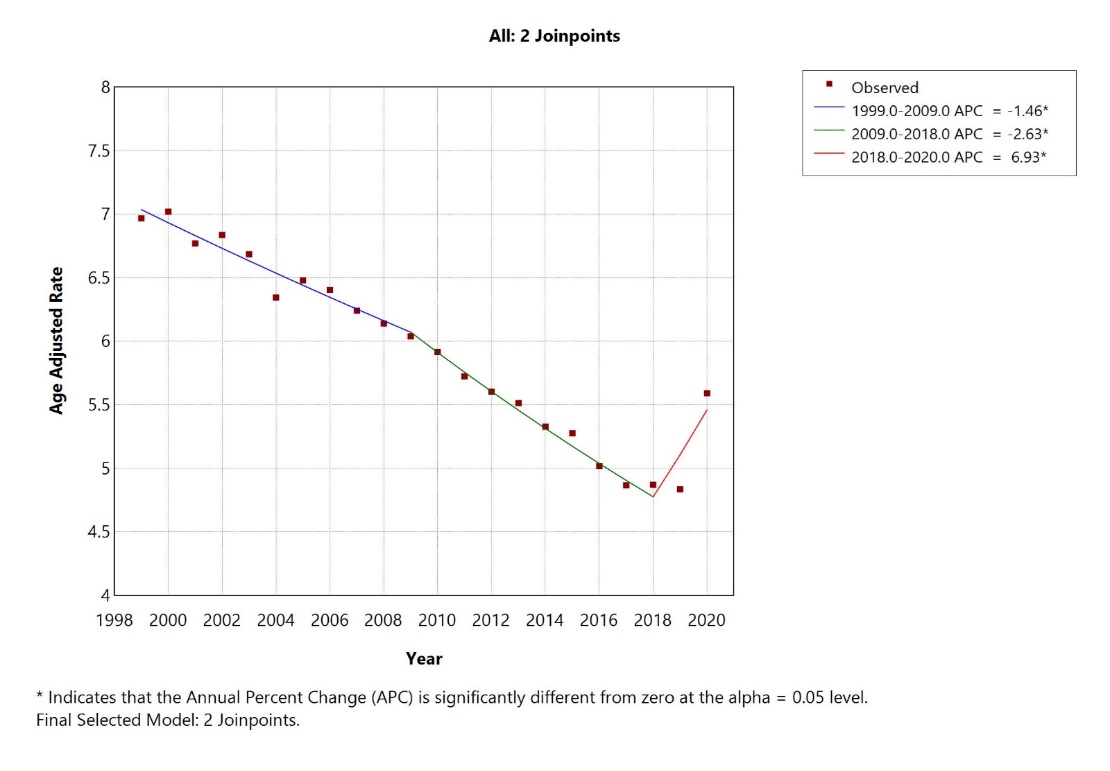

Supplement: Supplementary file 1 — Supplementary file. [file CLC-48-e70180-s001.docx]
